# A service at a breaking point: the NHS in the UK

**DOI:** 10.1016/j.eclinm.2024.102800

**Published:** 2024-08-27

**Authors:** eClinicalMedicine

On July 5, 1948, the UK established the first free at the point of access health-care system in western society: the National Health Service (NHS). The brainchild of Aneurin Bevan who served as Minister of Health in Clement Atlee's Labour government of 1945–51. 76 years later, and following a landslide election victory, Kier Starmer's Labour government must take action to secure the long-term future of a care service that has been left crippled by a decade of underfunding and scarred by a global pandemic, with staff physically and emotionally exhausted.

From 1997 to 2010 under the New Labour governments of Blair and Brown real-terms NHS funding increased by an average of 5.5% per year across the three terms. However, following the financial crash of 2008 and the actions of the Conservative-led austerity government NHS funding was cut to increases of 1.1% per year from 2010 to 2015. Moreover, from 2010 to 2019 health spending per patient in the UK was 18% lower than the average amount spent by the EU14 (the 14 member countries of the EU before 2004). OECD data from 2022 showed that the UK spends on average £3005.02 per person. Only four EU14 members spend less: Greece (£1756.82), Italy (£2517.68), Portugal (£2130.95), and Spain (£2369.18); Germany (£4186.20), Belgium (£3664.59), Austria (£3869.18), and France (£3644.55), all spend more. If spending had been increased to the EU14 average (£3655.00 per patient) an additional £40 billion per year would have been spent on patients and £33 billion more spent on health-care capital (eg, IT, equipment, and buildings). Although the average funding levels increased in response to the COVID-19 pandemic, 2 years of targeted funding to tackle a specific health threat has done little to address a decade of underfunding and resourcing.

Exacerbated by the COVID-19 pandemic, the number of people waiting for consultant-led care in the UK rose from 4.57 million in February, 2020, to 7.77 million in September, 2023. The most recent data show a small decrease to 7.60 million people in May, 2024, of whom 3.11 million had been waiting for more than 18 weeks. Cancer treatment wait times in the UK are a key example of the backlog faced. Although the number of patients diagnosed within 4 weeks is above the 75% target (76.4%), the percentage of patients who received first treatment within 1 month of a treatment decision (92%) and the percentage of patients who received first treatment within 2 months of an urgent referral (66%) are far below the set targets (96% and 85% respectively). A study published by *The Lancet Oncology* in March, 2024, shows the extent to which the UK lags behind other countries. Using pre-pandemic data, the average wait time to start chemotherapy was 48 days in England and 65 days in Scotland, but only 39 days in Norway. The differences were worse for the commencement of radiotherapy, with patients in the UK waiting between 53 (Northern Ireland) and 81 (Wales) days to start treatment compared with 42 days in Newfoundland, Canada. The extent of the challenge facing the NHS and the new government is also exemplified by the ongoing crisis in accident and emergency (A&E) departments. The number of people waiting more than 4 h to be admitted following presentation at A&E increased from 100,579 in January, 2020, to a peak of 170,283 in December, 2022, and a high this year of 158,721 in January, 2024. Of major concern is the increase in patients waiting 12 h before admission, which increased from a pre-pandemic peak of 2847 in January, 2022, to 54,532 in December, 2022. The number of patients waiting 12 h or more decreased to 38,106 in June 2024, but this still represents an 82-times increase on pre-pandemic levels during a month without the pressures a northern hemisphere winter brings to health-care systems.

In England alone there are an estimated 111,000 health-care job vacancies. These figures show the UK has 3.18 doctors per 1000 people, by comparison Germany has 4.3. The same is true for nurses. An OECD report stated that in 2021 there were 8.7 nurses per 1000 people in the UK, although this is better than Spain (6.3) and Portugal (7.4), the UK's nursing levels are lower than the average of the 37 OECD countries (9.2) and falls far below the number of nurses in Finland (18.9), Australia (12.8), and Ireland (12.7). In short, the UK is desperately short of health-care professionals. The number of vacancies within the NHS is exacerbated by the well documented recruitment issues caused by Brexit and an aging workforce that is choosing to retire earlier, with almost 25,000 doctors estimated to retire in the next 10 years. Moreover, the number of health-care professionals leaving the NHS due to health concerns (up 189%) or to improve work-life balance (up 163%) has grown substantially from 2013 to 2023. With 29% of staff reporting that they often think about leaving the NHS and one in five staff reporting bullying or harassment, issues regarding staff recruitment and retention and ensuring a healthy and safe working environment are deeply rooted.

Kier Starmer's promise to the country during the June 2023 election was one word: change. This seems to have been put into immediate action. On July 11, 2024, an independent review of the NHS was announced by Wes Streeting, Minister for Health and Social Care, to be led by Prof Lord Darzi. The aim of the investigation is to provide an expert understanding of the current state and performance of the NHS and outline the key challenges that the Labour government's 10-year plan for the health service will focus on. We welcome the expected focus on primary care. Darzi himself has previously reported that general practice requires life-saving surgery, and, with the government looking to divert billions of pounds to primary care and manifesto pledges to modernise booking systems and train thousands more GPs, it appears that there is political desire to revitalise this crucial health-care service. With the publication of the report expected in September, 2024, politicians, health-care professionals, and patients alike will not have to wait long for the diagnosis. The emphasis will then shift to the government to ensure that the recommendations from the Darzi report are fully implemented. We call on all political parties to commit to the recommendations of this independent assessment and ensure the best outcomes for patients.

It is beyond a doubt that frontline health-care staff work incredibly hard in the face of surmounting pressures and challenges; however, it is clear that the NHS has been stretched to near breaking point. In a 1948 address Bevin explained “the sooner we start, the sooner we can try together to see to these things and to secure the improvements we all want”, the time has come for this Labour government, riding a tidal wave of political capital, to support staff, protect the NHS, and save lives.

